# Developing and Validating a Breast Cancer Screening Health Literacy Questionnaire Guided by Sørensen’s Integrated Model for Malaysian Adults

**DOI:** 10.21315/mjms-10-2025-719

**Published:** 2026-02-28

**Authors:** Melissa Johari Chan, Nik Daliana Nik Farid, Nithiah Thangiah, Muhd Zulfadli Hafiz Ismail, Fairuz Abdul Rani, Siti Sarah Fadzil, Ruthashini R Selvasingam, Tan Cia Vei

**Affiliations:** 1 Department of Social and Preventive Medicine, Faculty of Medicine, Universiti Malaya, Kuala Lumpur, Malaysia; 2 Ministry of Health Malaysia, Wilayah Persekutuan Putrajaya, Malaysia; 3 Centre for Population Health, Department of Social and Preventive Medicine, Faculty of Medicine, Universiti Malaya, Kuala Lumpur, Malaysia; 4 Sector for Biostatistics and Data Repository, National Institutes of Health, Shah Alam, Selangor, Malaysia

**Keywords:** breast cancer, screening, health literacy, questionnaire development, psychometric validation, Sørensen’s Integrated Model

## Abstract

**Background:**

Health literacy is essential for informed decision-making and cancer prevention. In Malaysia, limited health literacy contributes to delayed breast cancer diagnoses and poorer outcomes among disadvantaged populations. However, there is no validated instrument for assessing breast cancer screening health literacy. Accordingly, this study aimed to develop and validate such a questionnaire for Malaysian adults.

**Methods:**

A three-phase, multi-method design was employed. Phase One involved item generation through a systematic review, online surveys, expert validation and translation. Phase Two included cognitive debriefing and an analysis of test–retest reliability. Phase Three consisted of a cross-sectional survey of 779 adults (mean age 36.6 years; 66% female) attending clinics in Selangor and Johor. The scale was evaluated psychometrically with exploratory factor analysis, internal consistency and confirmatory factor analysis using covariance-based structural equation modelling.

**Results:**

The initial 66-item tool was refined to a questionnaire with 41 items in seven domains showing strong reliability (Cronbach’s *α* ≥ 0.93; corrected item–total correlations ≥ 0.71). Weighted kappa values indicated moderate to almost perfect agreement (> 0.41) for most items. Confirmatory factor analysis showed factor loadings of 0.75 to 0.92, average variance extracted > 0.50, composite reliability > 0.70, heterotrait–monotrait ratios < 0.90 and acceptable fit indices (χ^2^/*df* = 2.18, GFI = 0.85, AGFI = 0.83, CFI = 0.95, TLI = 0.95, RMSEA = 0.052).

**Conclusion:**

The validated 41-item Breast Cancer Screening Health Literacy Questionnaire is a reliable and culturally appropriate tool in Bahasa Malaysia that can be employed in clinical and community settings to identify literacy gaps, develop tailored health promotion strategies and strengthen early detection initiatives.

## Introduction

Health literacy (HL) is a key determinant of health outcomes that reflects individuals’ ability to obtain, process and apply information for informed decision-making. Beyond basic reading, HL encompasses the interactive and critical competencies underlying engagement with healthcare and preventive practices. Limited HL is consistently associated with poor health behaviours, inadequate disease management, delayed diagnoses and higher morbidity and mortality (1–3).

In Malaysia, breast cancer is the most common form of cancer among women and a major cause of cancer-related deaths (4). Mortality and the treatment burden continue to be high because of late-stage presentation (4). Despite the proven role of screening in early detection, uptake remains low, particularly among socioeconomically disadvantaged and rural groups (5–7), due to cost, cultural beliefs, low awareness and navigation challenges within healthcare (8, 9). HL is a crucial yet underexplored factor influencing knowledge, self-efficacy and motivation for screening participation.

Current HL and cancer literacy instruments are largely for a general context or were developed for Western contexts, which limits their applicability in Malaysia (10–12). Many focus on knowledge or functional literacy while overlooking appraisal, application and motivational aspects and none have been validated in Bahasa Malaysia, the national language and primary medium of health communication in the country, thereby limiting accessibility and cultural relevance.

Sørensen’s Integrated Model provides a comprehensive framework comprising four competencies–those of accessing, understanding, appraising and applying health information – across the healthcare, disease prevention and health promotion domains (2). It also treats knowledge, motivation and competence as essential to informed decision-making. Tools like the European Health Literacy Survey Questionnaire (HLS-EU-Q) highlight the importance of a multidimensional HL approach (13), yet none specific to breast cancer screening exists for Malaysia.

This study developed and validated a Breast Cancer Screening HL Questionnaire (BCSHLQ) in Bahasa Malaysia guided by Sørensen’s Integrated Model to address this gap. This instrument was designed to identify literacy needs, inform culturally appropriate educational strategies and guide interventions to enhance screening uptake. It also facilitates the evaluation of health promotion efforts and policies aimed at reducing disparities in breast cancer outcomes.

## Methods

### Study Design and Phases

This cross-sectional validation study was conducted in three phases: item generation (Phase One), scale development (Phase Two) and scale evaluation (Phase Three). A multi-method approach in which qualitative and quantitative techniques were combined was employed to ensure methodological rigour.

### Phase One: Item Generation

Item generation, guided by Sørensen’s Integrated Model, combined deductive and inductive approaches. The deductive approach involved a systematic review of current breast cancer HL instruments, while the inductive approach included an online survey of Malaysian adults and expert content validation

The systematic review followed Preferred Reporting Items for Systematic Reviews and Meta-Analyses (PRISMA) guidelines to identify relevant constructs and items from established questionnaires to inform item development (14). Searches were conducted across four databases (Ovid Medline, ScienceDirect, CINAHL and Web of Science), incorporating Medical Subject Headings terms in the Boolean string: (‘breast’) AND (‘neoplasms’ OR ‘cancer’) AND (‘health literacy’). Eligibility criteria were English-language instruments published up to December 2023 reporting on the development or adaptation of breast cancer HL instruments were included. Grey literature and inaccessible full texts were excluded. Two reviewers independently screened the titles and abstracts and resolved discrepancies through discussion or assessment by a third reviewer. Data extraction and quality assessment were undertaken to identify high-quality items and domains relevant to breast cancer screening HL.

An online survey was administered using Google Forms to capture the perspectives of Malaysian adults on breast cancer screening. We used snowball sampling in which the first respondents were invited to complete and then share the survey. The survey collected sociodemographic details and included six open-ended questions on respondents’ awareness of screening, reasons for seeking information, sources of information, challenges in accessing information and barriers to screening participation. Responses were thematically analysed and mapped to HL domains to inform item generation. Eligible participants were Malaysian adults aged 18 years and above who were proficient in either Malay or English.

Expert content validation was conducted with five panellists: a breast surgeon, public health specialist, family medicine specialist, a nurse with doctoral training and research experience in breast cancer and a representative of a non-governmental organisation targeting breast cancer, each with a minimum of five years of experience. The experts rated the candidate items for relevance and clarity on a four-point scale. The Content Validity Index (CVI) was calculated at the item (I-CVI) and scale (S-CVI/Ave) levels, with thresholds of 1.0 and ≥ 0.90, respectively (15). Feedback was incorporated until consensus was reached.

The questionnaire was translated into Bahasa Malaysia using a forward–backward translation procedure in which two independent bilingual Malay translators conducted the forward translation, one medically trained with formal translation training, the other a professional medical and pharmaceutical translator with more than five years of experience with health-related materials. Both were briefed on the questionnaire’s objectives. A review committee consisting of the translators and study researchers reconciled the discrepancies to produce a harmonised Malay version. Two independent native Englishspeaking bilingual translators experienced in academic and technical translation then performed backward translation. The review committee compared the backward translations with the original English version and discrepancies were resolved by consensus until conceptual and linguistic equivalence was attained.

### Phase Two: Scale Development

This phase involved pre-testing through cognitive debriefing and test–retest reliability. Cognitive debriefing was conducted with 10 participants recruited from Klinik Kesihatan Sungai Besi, Kuala Lumpur (16), who were selected purposively to ensure variation in age, gender, ethnicity and educational background, based on predefined inclusion and exclusion criteria. The cognitive debriefing employed a verbal probing technique after the participants completed the questionnaire. Sessions were conducted in groups of three to four participants and were guided by a semi-structured set of questions to ensure consistency across groups. The probes were used to explore participants’ understanding of items’ wording, clarity of meaning and cultural appropriateness. Participant feedback was reviewed and grouped by issues such as unclear wording, ambiguity and cultural appropriateness and used to guide item rewording and refinement.

Test–retest reliability was evaluated with 30 participants from the Department of Social and Preventive Medicine, Universiti Malaya, following recommended stability testing protocols (17). Participants completed the questionnaire twice at a two-week interval. Reliability was assessed using quadratic weighted kappa statistics, with values > 0.40 indicating acceptable reliability (18). Data collection for Phase Two was conducted between July and September 2024.

### Phase Three: Scale Evaluation

A large-scale field study was conducted from October to December 2024 to evaluate the psychometric properties of the BCSHLQ. Data were collected from two public health clinics in Selangor, two in Johor and one private clinic in Selangor, purposely selected to represent urban and rural populations in high-burden states. The particular sites were chosen based on patient volume and feasibility for achieving the required sample size within the data collection period and ensuring diverse sociodemographic representation.

Purposive sampling was applied in Phases Two and Three using consistent inclusion and exclusion criteria. Eligible participants were Malaysian citizens aged 18 years and above and proficient in Malay. Individuals who declined participation or were mentally or physically unable to respond accurately were excluded.

The sample size in Phase Three was guided by psychometric recommendations. At least 300 participants or a 5:1 subject-to-item were recommended for exploratory factor analysis (EFA) (19), and 200 to 500 for confirmatory factor analysis (CFA), depending on model complexity (20). A minimum of 500 participants was targeted to ensure stable estimates.

### Analytical Approach

Analyses were conducted using IBM SPSS Statistics 29.0 and AMOS 29.0. Data from the large-scale field study were randomly split into two independent sub-samples using the random case selection function in SPSS, with one sub-sample (*n* = 390) used for EFA and internal consistency analysis to identify the underlying factor structure and the other (*n* = 389) reserved for CFA to assess the stability of the measurement model. This split-sample approach enables the internal cross-validation of the factor structure within a single study and is widely recommended in psychometric scale development to reduce the risk of overfitting when EFA and CFA are conducted sequentially (20, 21).

Multivariate normality was assessed using Mardia’s multivariate kurtosis test, where the standard threshold values > 7.00 for kurtosis and > 1.96 for the critical ratio are taken as indicating violations of normality. Where these thresholds were violated, bootstrapping with 500 samples and 95% bias-corrected confidence intervals was applied to ensure robust parameter estimation (22). Common method variance was evaluated using Harman’s single-factor test, with > 50% variance explained indicating potential bias (23).

EFA using principal axis factoring with Promax rotation was employed to examine the underlying structure. Sampling adequacy was assessed using the Kaiser-Meyer-Olkin (KMO) statistic (≥ 0.70) (20) and Bartlett’s test of sphericity (*P* < 0.05) (24). Domains were retained based on eigenvalues > 1 and visual inspection of Cattell’s scree plot (25), and items were retained if factor loadings were ≥ 0.40 (20) with cross-loading differences ≥ 0.15 (21). Internal consistency was assessed using Cronbach’s alpha (≥ 0.70) (26) and corrected item–total correlations (CITCs; ≥ 0.30) (27).

CFA was performed using covariance-based structural equation modelling (CB-SEM) to confirm the measurement model. Model fit was evaluated using the thresholds χ^2^/*df* < 5.00, RMSEA < 0.10, GFI ≥ 0.90 (≥ 0.85 acceptable in scale development) (28, 29), AGFI ≥ 0.90 (≥ 0.80 acceptable in scale development) (28, 29), and comparative fit index (CFI) and TLI ≥ 0.90 (30). Construct validity was assessed through convergent validity (average variance extracted, AVE ≥ 0.50), composite reliability (CR ≥ 0.60) (30), and discriminant validity (heterotrait– monotrait ratio [HTMT] < 0.90) (31). Where model fit was sub-optimal, refinement was guided by modification indices (> 15) (30). The error terms of redundant item pairs within the same domain were allowed to correlate as free parameters, where theoretically justified to improve model fit (30) further.

## Results

### Systematic Review

**Figure 1:**
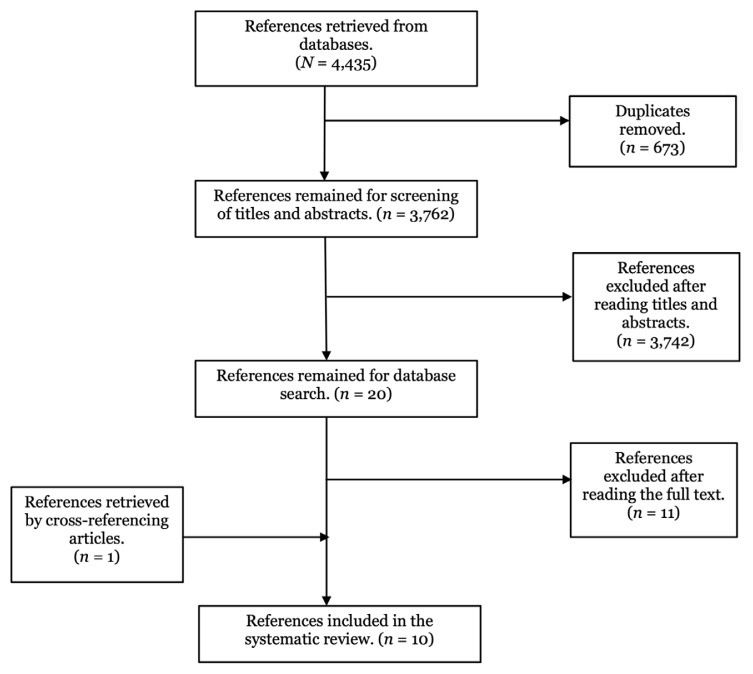
PRISMA flow diagram of study selection

The database search yielded 4,435 records, from which 673 duplicate records were removed. After screening the remaining 3,762 titles, 20 full texts were reviewed and one additional study was identified through cross-referencing. Ten studies met the inclusion criteria. They originated from North America (*n* = 5), Asia (*n* = 2), Europe (*n* = 2) and Oceania (*n* = 1) and involved populations such as breast cancer patients, at-risk women, students and community health workers, with sample sizes ranging from 16 to 1,306 participants. The theoretical frameworks employed included Nutbeam’s model, Baker’s conceptualisation and Sørensen’s Integrated Model. The most commonly used psychometric tests were Cronbach’s alpha, EFA and CFA. Four studies were rated as having high-quality, four medium and two poor. Figure 1 presents the PRISMA flow and Table 1 summarises the included studies. Detailed quality ratings are provided in Supplementary File S1.

### Online Public Survey

A total of 117 Malaysian adults participated. Most were female (74.4%), Chinese (42.7%), aged 31 to 40 years (47.9%), married (62.4%), tertiary-educated (87.2%) and urban residents (89.7%). Their survey responses revealed the themes of screening awareness, perceived importance, information sources and barriers like cost, stigma and misinformation, which were mapped to Sørensen’s model to inform item generation. The thematic mapping is presented in Supplementary File S2.

### Content Validation

Two rounds of content validation were conducted. In Round One, 12 items in Sections A, E and G had values of I-CVI ≤ 1.0, and the value of S-CVI/Ave was 0.82, below the 0.90 threshold for relevance. Regarding clarity, 18 items were rated as ambiguous, excessively technical and culturally unsuitable. Revisions improved relevance and clarity, reducing the pool to 66 items. In Round Two, I-CVI = 1.0 for all items and S-CVI/Ave improved to 1.0. The experts unanimously agreed that the items were concise, unambiguous and culturally appropriate.

### Translation

Forward–backward translation produced a harmonised Malay version requiring only minor semantic adjustments. This finalised instrument was used in the study.

### Cognitive Debriefing and Test–Retest Reliability

Cognitive debriefing of 10 adults (80.0% female; median age of 33 years, IQR 26.8–38.5) confirmed that the layout and instructions were clear, though several issues were identified. Items with statistical expressions (A8, B10, C9, D9, F9) were misinterpreted as containing question numbers or response scale points rather than risk estimates. Item D5 was interpreted as directional guidance rather than as identifying screening centres and G7 (“mengakses perkhidmatan”) was associated with Internet use rather than health service access. Terms such as *gejala* (E9), *rintangan* (G10) and “mengatur ujian saringan” (F5) were considered unfamiliar.

Revisions were made accordingly: statistical items were clarified with contextual phrases, *gejala* was replaced with *simptom* and *rintangan* with *halangan* and Item F5 was revised to “mengatur janji temu ujian saringan.” A standardised explanation was added to clarify the intended frame of reference. Completion times (11 to 25 minutes) were acceptable and no respondents reported fatigue. The sociodemographic characteristics of the respondents are presented in Supplementary File S3.

Test–retest reliability measurements among 30 adults (66.7% female, median age of 23 years, IQR 22.0–37.3) showed moderate (κ = 0.42–0.60), substantial (0.61–0.77) or almost perfect (≥ 0.80) agreement. One knowledge item (E3; κ = 0.23) showed only fair reliability and was removed. The sociodemographic characteristics of the respondents are provided in Supplementary Files S4.

### Large-Scale Field Study

#### Respondent Characteristics

Of the 793 responses received, 779 were complete. Participants were predominantly female (65.9%), with a mean age of 36.6 years (SD = 13.4). Most were Malay (71.1%), married (57.0%), tertiary-educated (51.6%), employed (80.2%) and urban residents (54.2%). Table 2 summarises respondent characteristics.

#### Exploratory Factor Analysis

**Figure 2:**
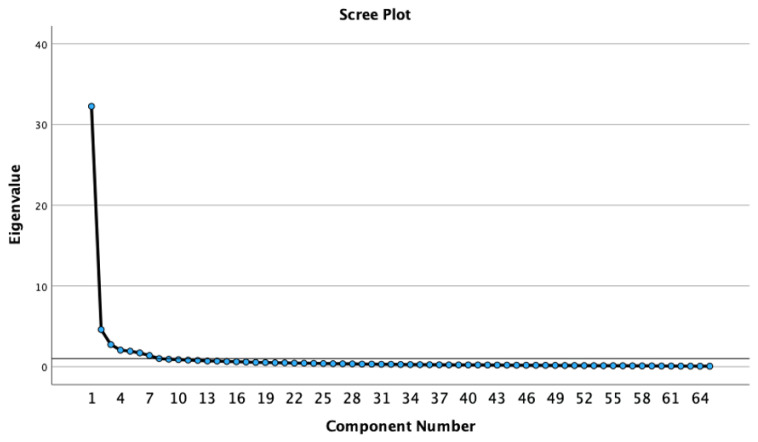
Scree plot from exploratory factor analysis showing the seven domains extracted

The data of 390 participants (KMO = 0.94; Bartlett’s χ^2^ = 18,545.23, *P* < 0.001) supported factorability. Principal axis factoring with Promax rotation extracted seven factors, consistent with the guiding framework. Items with factor loadings < 0.40 or cross-loading differences < 0.15 were removed, yielding a 61-item solution (range 0.51–0.89). The scree plot (Figure 2) demonstrated a clear point of inflection after the seventh factor and all retained factors had eigenvalues greater than 1.0, supporting a seven-domain solution under the Kaiser criterion. This structure was in alignment with Sørensen’s Integrated Model of HL and its extension by including knowledge, competence and motivation as distinct but related dimensions of breast cancer screening HL.

#### Internal Consistency

Cronbach’s alpha values ranged from 0.93 to 0.96 and CITC values from 0.71 to 0.89 across domains, indicating strong reliability.

#### Confirmatory Factor Analysis

The data for the other 389 participants were used for CFA. The initial model showed sub-optimal fit. Stepwise refinement guided by modification indices (MI) removed 19 items and correlated 11 residuals, producing a 41-item model across seven domains. The refined model demonstrated satisfactory fit (χ^2^/*df* = 2.18, GFI = 0.85, AGFI = 0.83, CFI = 0.95, TLI = 0.95, RMSEA = 0.052). The Goodness-of-Fit Index (GFI) and Adjusted Goodness-of-Fit Index (AGFI) values, though below 0.90, were acceptable (≥ 0.85 and ≥ 0.80) for scale development. The standardised factor loadings (0.75–0.92) confirmed unidimensionality and the values of AVE ≥ 0.50 and CR = 0.87–0.95 supported convergent validity and internal consistency. Finally, the HTMT value < 0.90 confirmed discriminant validity.

#### Final Model

**Figure 3:**
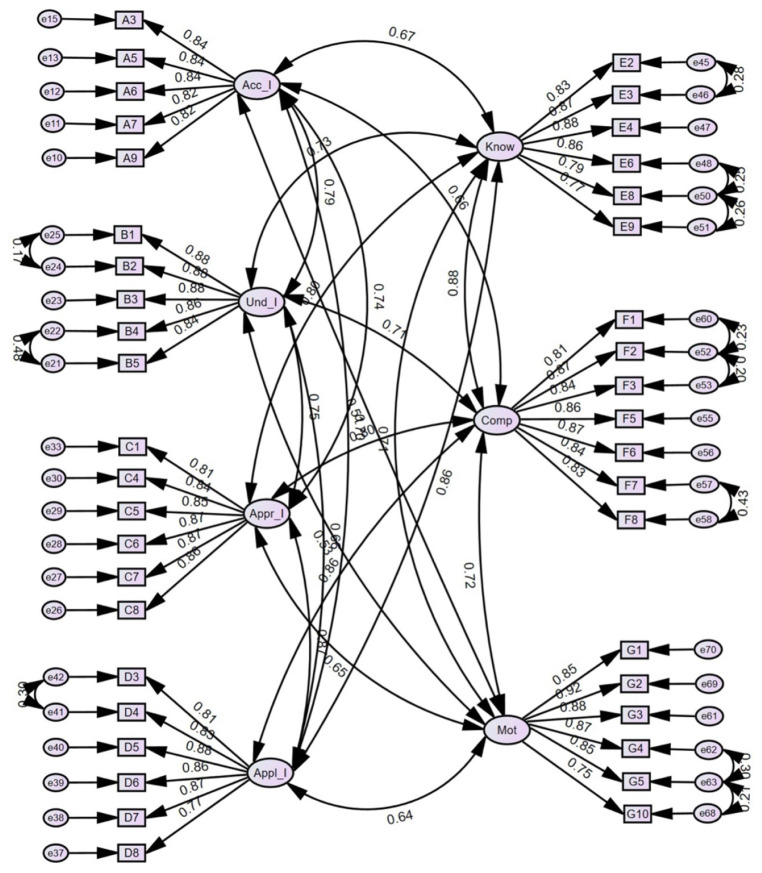
Final Seven-Domain Confirmatory Factor Analysis Model of the BCSHLQ Acc_I = access information; Und_I = understand information; Appr_I = appraise information; Appl_I = apply information; Know = knowledge; Comp = competence; Mot = motivation

The validated BCSHLQ in Bahasa Malaysia comprises 41 items across seven domains, reflecting a multidimensional structure consistent with Sørensen’s Integrated Model. The instrument captures the cognitive (Access, Understand, Appraise, Apply Information), knowledge, competence and motivational dimensions of screening literacy. The final model is illustrated in Figure 3.

## Discussion

This study is the first to develop and validate a version of the BCSHLQ that is culturally appropriate for Malaysia. Guided by Sørensen’s Integrated Model, this instrument has a robust seven-domain structure supported by exploratory and confirmatory factor analyses. Psychometric testing confirmed strong reliability and validity, showing the BCSHLQ to be both conceptually comprehensive and methodologically sound.

### Comparison with the Literature

The BCSHLQ extends previous HL measurement efforts. The Cancer HL Test (CHLT-30 and CHLT-6) of Dumenci et al. (10) showed solid psychometric results, but it is focused primarily on functional literacy within general cancer populations. Similarly, Barros et al. (35) translated the CHLT into Portuguese, but its psychometric quality is modest, limiting its utility beyond preliminary testing. Shan and Ji’s (36) simplified Chinese version of the Breast Cancer Literacy Assessment Tool (C-B-CLAT) targets students, a narrow focus that limits its comprehensiveness.

Han et al.’s (37) Assessment of HL in Cancer Screening (AHL-C), guided by Baker’s conceptualisation for Korean American women, is psychometrically strong but population-specific and contains only four sub-scales (print literacy, numeracy, comprehension and familiarity). Gibbs et al. (38) piloted a nutrition literacy tool adapted for breast cancer patients, while Huang et al. (39) validated the HLS-EU-Q in Taiwanese women with breast cancer, closely following Sørensen’s framework; however, the higher-order domains were collapsed into a single factor, limiting multidimensional interpretation.

The present study operationalises Sørensen’s model into seven empirically supported domains integrating cognitive, functional and motivational factors. Unlike previous tools, the BCSHLQ offers a comprehensive measure of screening literacy tailored to the Malaysian context that combines conceptual depth with strong factorial structure and reliability.

### Theoretical and Public Health Significance

From a theoretical perspective, this study extends Sørensen’s framework to breast cancer screening, illustrating the importance of including motivation, competence and knowledge alongside the four core competencies. This multidimensional structure captures the broader skills necessary for informed screening decisions, beyond narrow measures of knowledge or functional literacy.

From a public health perspective, the BCSHLQ addresses urgent national needs. Breast cancer remains the most common cancer among Malaysian women, with late-stage diagnosis driving mortality (40). Despite decades of awareness campaigns, policy reviews reveal fragmented screening promotion and a limited integration of HL principles (41, 42). These findings align with Malaysia’s National Strategic Plan for Cancer Control Programme (NSPCCP) 2021–2025, which identifies strengthening early detection, improving health-seeking behaviour and enhancing cancer HL as key national priorities (43). Proposals for risk-stratified screening further highlight the need to address literacy-related barriers to ensure equitable participation (44).

Local studies have largely examined respondents’ knowledge, attitudes and practices, focusing on stigma, cultural beliefs and low awareness (45, 46), and thus fail to capture the broader competencies essential for screening engagement. The absence of validated HL tools has constrained both research and programme design in Malaysia.

Although the 2019 National Health and Morbidity Survey (NHMS) assessed general HL using the HLS-SF12, breast cancer screening literacy remains unmeasured at the national level (47). The BCSHLQ therefore, fills a critical gap and could be incorporated into national data platforms like the NHMS or linked initiatives like the National Cancer Registry to monitor screening literacy over time. It may also complement digital and community-based interventions to strengthen preventive behaviours and communication strategies.

International evidence reinforces the centrality of HL: low literacy is associated with misperceptions of risk and greater perceived barriers (48), while literacy-focused interventions have been shown to improve self-care (49) and preventive practices (50). By introducing the first validated breast cancer screening HL tool suited to the context of Malaysian society, this study provides researchers, clinicians and policymakers with a culturally grounded instrument to identify literacy needs, design targeted interventions and evaluate outcomes; the BCSHLQ can be employed in equitable, risk-stratified screening and early detection policies that are responsive to population HL levels.

### Strengths and Limitations

The main strength of this study lies in its rigorous multi-method design encompassing systematic review, survey, expert validation, translation, cognitive debriefing, test–retest assessment and psychometric evaluation. Guided by Sørensen’s Integrated Model and informed by both local and global evidence, the BCSHLQ demonstrates conceptual relevance and cultural fit. Its seven domains include not only functional literacy but also knowledge, motivational and competence-based dimensions often overlooked in current tools.

Several limitations should be acknowledged. The use of purposive sampling from selected clinics may limit generalisability. Although EFA and CFA were conducted on randomly split sub-samples of a single data set, this approach does not fully replace validation using an independent external sample. Future studies should therefore evaluate the BCSHLQ in independent populations and settings further to confirm the stability and generalisability of the factor structure. In addition, although responses were anonymous, the self-administered format might have introduced social desirability bias, whereby participants gave favourable rather than accurate responses. This risk was mitigated through careful item phrasing and assurances of confidentiality. While Harman’s single-factor test suggested minimal common method bias, procedural controls (expert validation, cognitive debriefing and anonymous administration) and statistical remedies (bootstrapping and CFA diagnostics) further reduced potential effects. Moreover, the questionnaire was validated only in Bahasa Malaysia; translation into other major languages (English, Mandarin, Tamil) would enhance inclusivity. Future studies should validate the BCSHLQ across more diverse populations, including underserved groups such as the Orang Asli and across different regions to ensure national representativeness. Research should also establish population-level cutoffs (e.g., inadequate, problematic, sufficient, excellent) to support its use in clinical and policy settings.

## Conclusion

This study developed and validated the first BCSHLQ in Malaysia, guided by Sørensen’s Integrated Model of HL. This seven-domain instrument, which demonstrates strong reliability, validity and cultural relevance, addresses a critical gap in instruments to measure literacy related to breast cancer screening by providing researchers, clinicians and policymakers with a practical tool to identify literacy needs and design targeted interventions, which can support equitable participation in national screening initiatives and promote earlier detection. Future research should validate the instrument across languages and regions and evaluate its application in intervention and policy studies. Ultimately, the BCSHLQ constitutes a foundation for literacy-driven cancer prevention strategies, contributing to Malaysia’s long-term goal of reducing disparities in early detection and empowering individuals to take informed action for their health.

## Supplementary Information



## Figures and Tables

**Table 1:** Characteristics of included studies in the systematic review

Author(s)	Study aim	Sample Context No. of domains	Framework	FA	Internal consistency (reliability)
Shan and Ji (33)	To translate, culturally adapt and validate the Breast Cancer Literacy Assessment Tool (B-CLAT) into simplified Chinese (C-B-CLAT) for use among Chinese college students	50 female students in China Functional HL specific to breast cancer 3 domains	Not specified; adapted from B-CLAT, which measures functional HL	None	Cronbach’s alpha (0.61)
Han et al. (34)	To develop and validate the Assessment of Health Literacy in Cancer Screening (AHL-C) for breast and cervical cancer screening among Korean American women and to evaluate its sensitivity to changes following a literacy-focused intervention	560 Korean American women from the community Reading fluency and prior knowledge 4 domains	Baker’s conceptualisation of HL	None	Cronbach’s alpha (0.96)
Gibbs et al. (35)	To adapt the Nutrition Literacy Assessment Instrument for breast cancer patients, pilot test its validity and reliability and evaluate sensitivity to intervention effects	17 high-risk women and 73 breast cancer patients in the US Functional literacy, conceptual knowledge and applied skills 6 domains	Did not specify. Based on the author’s previous Nutrition Literacy Assessment Instrument (NLit) framework	CFA	Composite reliability (reported qualitatively)
Huang et al. (36)	To validate the reliability and validity of the HLS-EU-Q among Taiwanese women with breast cancer	475 women with breast cancer in Taiwan HL in relation to breast cancer 12 sub-domains	Sørensen’s integrated model of HL	CFA	Cronbach’s alpha (0.97)
Dumenci et al. (10)	To develop and validate the Cancer Health Literacy Test (CHLT-30 and CHLT-6) to measure and screen for cancer health literacy	1,306 adult Black and Hispanic cancer patients (16.3% with breast cancer) in the US Functional cancer HL Unidimensional	Did not specify	EFA/CFA	Cronbach’s alpha (0.88)
McDonald et al. (32)	To adapt a HL measure for adolescents and young adults with cancer and conduct initial validation	105 Australian Adolescents and young adults from a national cancer support organisation (< 21% with breast cancer) Functional, communicative and critical HL 3 domains	Nutbeam’s Model	EFA	Cronbach’s alpha (0.63–0.85)
Barros et al. (32)	To translate, culturally adapt and pre-test the Cancer Health Literacy Test (CHLT) for Portuguese cancer patients	71 cancer patients (17% are breast cancer patients) in Portugal Functional cancer HL Unidimensional	Based on the Cancer Health Literacy Test (CHLT-30)	None	Cronbach’s alpha (0.80–0.86)
Williams et al. (33)	To develop an orally administered breast and cervical cancer literacy tool for laypersons	16 community health workers in the US Functional HL specific to breast and or cervical cancer 3 domains	Did not specify	None	Cronbach’s alpha (0.85–0.91)
van der Vaart et al. (11)	To validate the Dutch version of Ishikawa’s functional, communicative and critical HL scale	209 with rheumatism, 79 women with breast cancer, 18 with rheumatoid arthritis in the Netherlands Functional, communicative and critical HL 3 domains	Nutbeam’s Model	CFA	Cronbach’s alpha (0.78–0.94)
Williams et al. (34)	To evaluate the reliability and validity of a revised Breast Cancer Literacy Assessment Tool (Breast-CLAT) for measuring functional understanding of breast cancer in English, Spanish and Arabic	543 women from Black, Latina and Arab American groups recruited through community health programmes Functional HL 3 domains	Did not specify	CFA	Cronbach’s alpha (0.73)

FA = factor analysis; EFA = exploratory factor analysis; CFA = confirmatory factor analysis; HL = health literacy

**Table 2:** Sociodemographic characteristics of respondents in the large-scale field study (*N* = 779)

Variable	*n* (%)
**Gender**	

Male	266 (34.1)
Female	513 (65.9)

**Age (years)**	

Mean (SD)	36.6 (13.4)
Min	18
Max	80

**Ethnicity**	

Malay	554 (71.1)
Chinese	127 (16.3)
Indian	58 (7.4)
Others	40 (5.1)

**Marital status**	

Single/unmarried	286 (36.7)
Married	444 (57.0)
Divorced	24 (3.1)
Widowed	25 (3.2)

**Highest education level**	

No formal education	12 (1.5)
Primary	26 (3.3)
Secondary	339 (43.5)
Tertiary	402 (51.6)

**Current employment status**	

Unemployed	95 (12.2)
Employed	625 (80.2)
Retiree	20 (2.6)
Student	39 (5.0)

**Employment sector**	

Government/semi-government	126 (16.2)
Private	476 (61.1)
Self-employed	32 (4.1)
Unemployed	145 (18.6)

**Monthly household income (RM)**	

< 2,500	269 (34.5)
2,500 to 3,169	148 (19.0)
3,170 to 3,969	71 (9.1)
3,970 to 4,849	54 (6.9)
4,850 to 5,879	41 (5.3)
5,880 to 7,099	47 (6.0)
7,100 to 8,699	29 (3.7)
8,700 to 10,959	37 (4.7)
10,960 to 15,039	34 (4.4)
> 15,039	49 (6.3)

**Area of residence**	

Rural	357 (45.8)
Urban	422 (54.2)
