# Supplementary material for: Developing and Validating a Breast Cancer Screening Health Literacy Questionnaire Guided by Sørensen’s Integrated Model for Malaysian Adults
Source: Malays J Med Sci. 2026 Feb 28;33(1):129–44. doi: 10.21315/mjms-10-2025-719 (PMC13471903; doi:10.21315/mjms-10-2025-719)
Supplement: Supplementary file 1 [file MJMS33(1)_Art11_SupplementaryFiles.pdf]

Supplementary File S1. Quality Ratings of Included Studies in the Systematic Review

Study	Followed a priori explicit theoretical framework	Reported efforts towards content validity	Structural validity through EFA	Internal construct validity	External construct validity	Reliability scores above 0.7	Total score	Interpretation (≤2=poor quality, 3-4=medium quality, 5-6 = high quality)
Shan & Ji (2023)	0	1	0	0	0	1	2	Poor quality
Han et al. (2014)	1	1	0	0	1	1	4	Medium quality
Gibbs et al. (2016)	0	1	1	1	1	1	5	High quality
Huang et al. (2018)	1	1	0	1	1	1	5	High quality
Dumenci et al. (2014)	0	1	1	1	1	1	5	High quality
McDonald et al. (2016)	1	0	1	0	0	1	3	Medium quality
Barros et al. (2022)	0	1	0	0	0	1	2	Poor quality
Williams et al. (2008)	0	1	0	0	1	1	3	Medium quality
van der Vaart et al. (2012)	1	1	0	1	1	1	5	High quality
Williams et al. (2013)	0	1	0	1	1	1	4	Medium quality

Scoring system: 0 = absent; 1 = present

Supplementary File S2. Thematic Mapping of Online Survey Responses for Item Generation

Question 1. What do you understand about breast cancer screening?

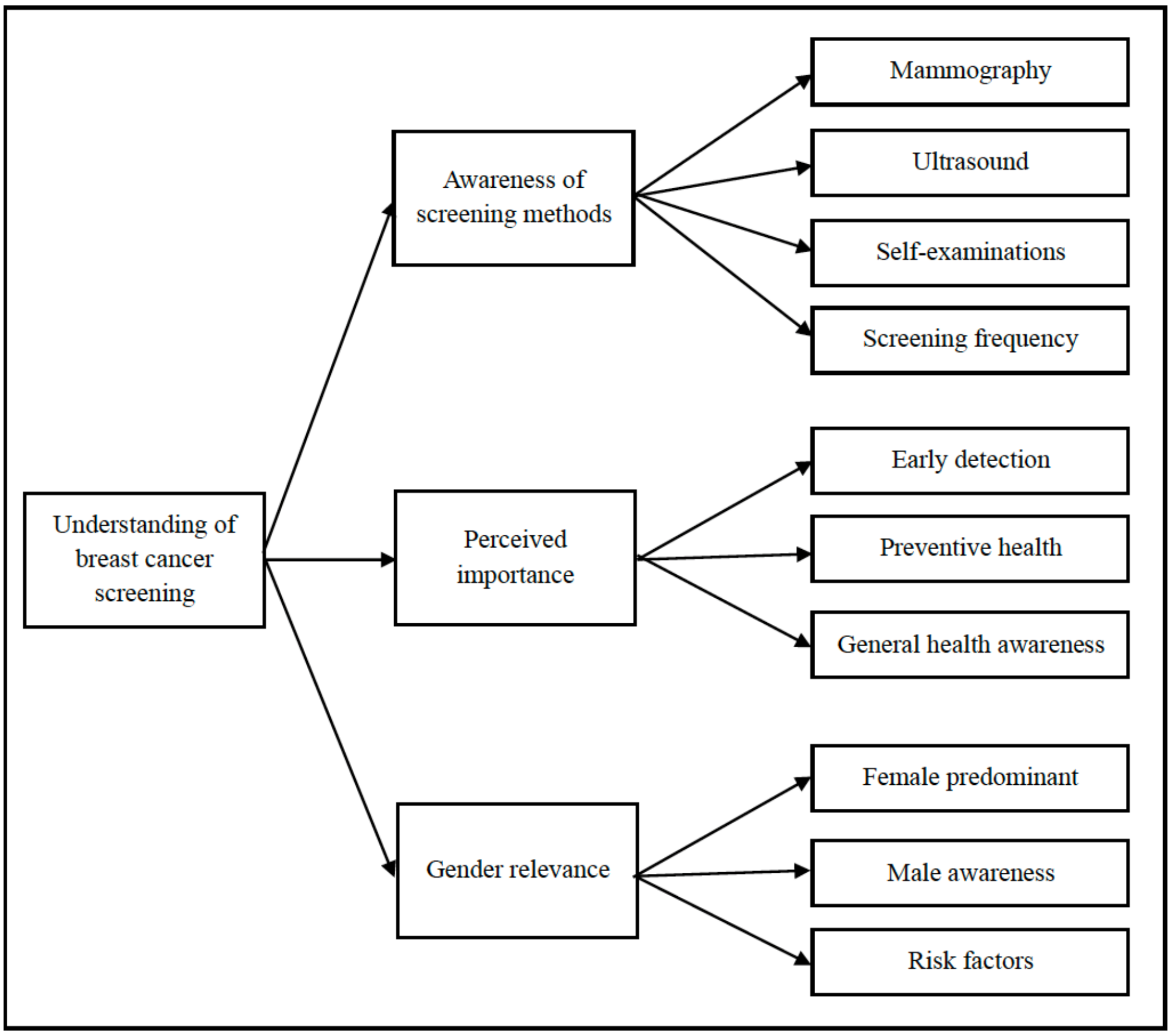

Question 2. Why do you think people seek information about breast cancer screening?

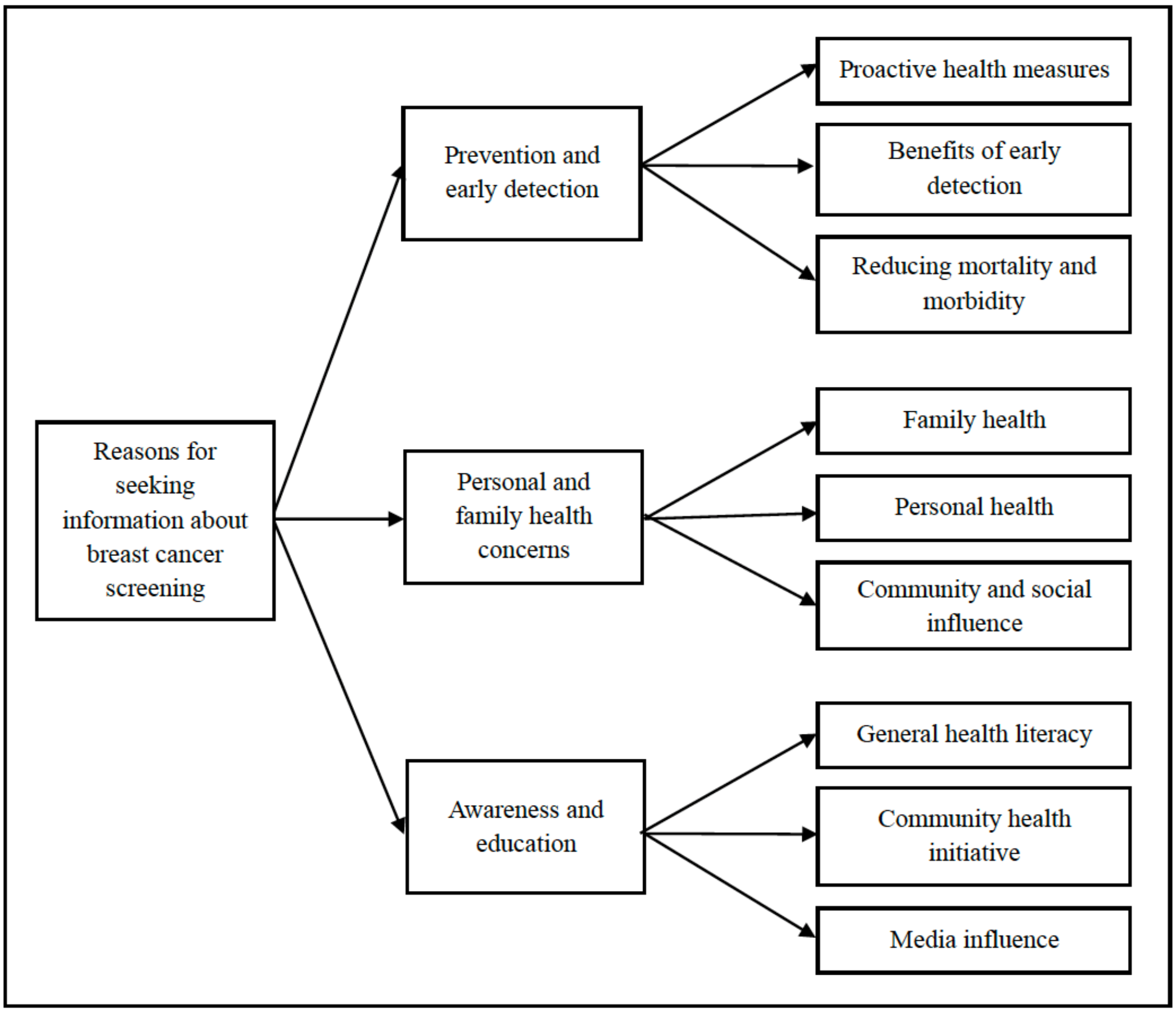

Question 3. Where do people usually seek information about breast cancer screening?

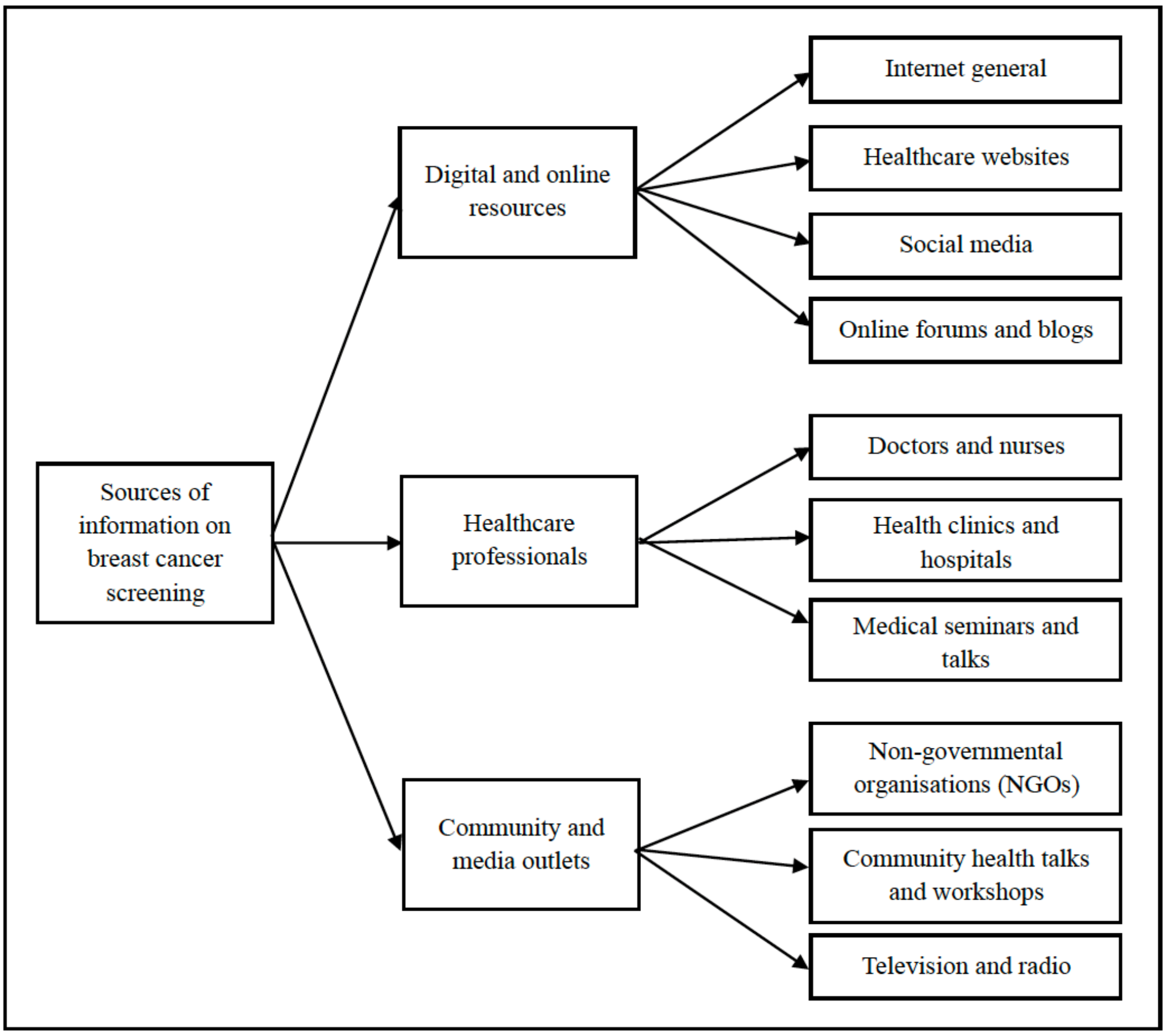

Question 4. What challenges do you think people face when seeking information about breast cancer screening?

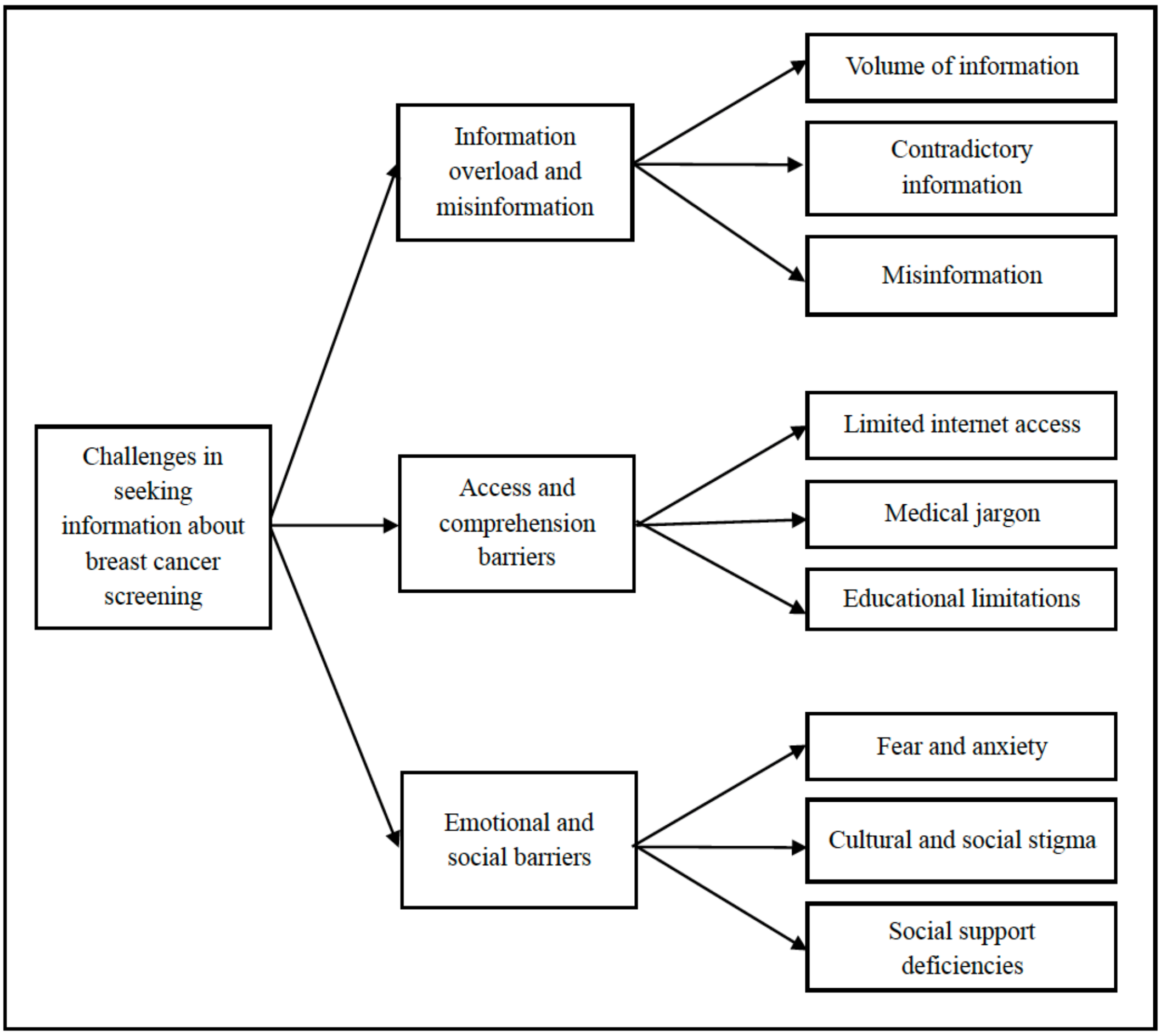

Question 5. Where do people typically go for breast cancer screening?

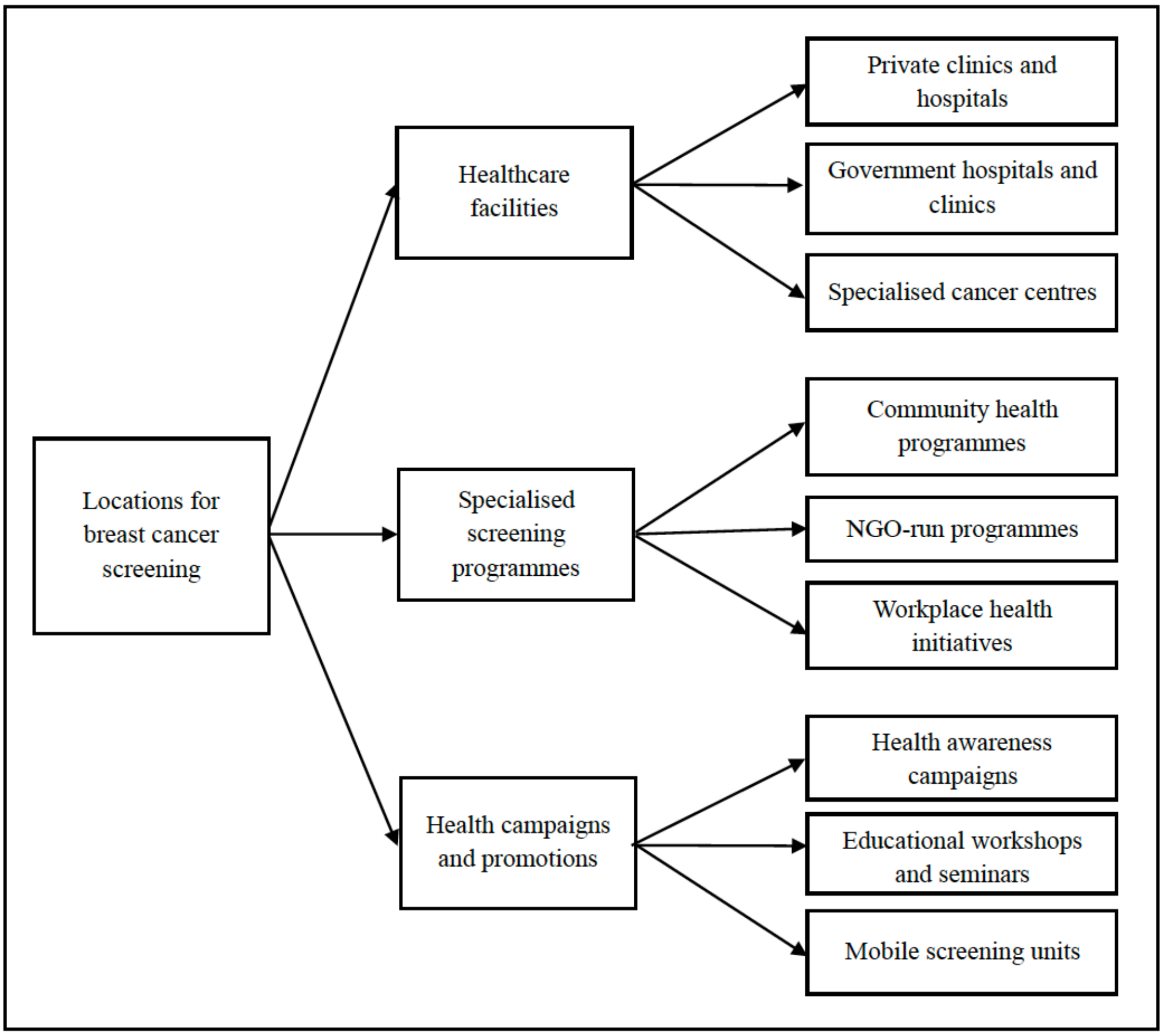

Question 6. What challenges do you think people face when trying to get screened for breast cancer?

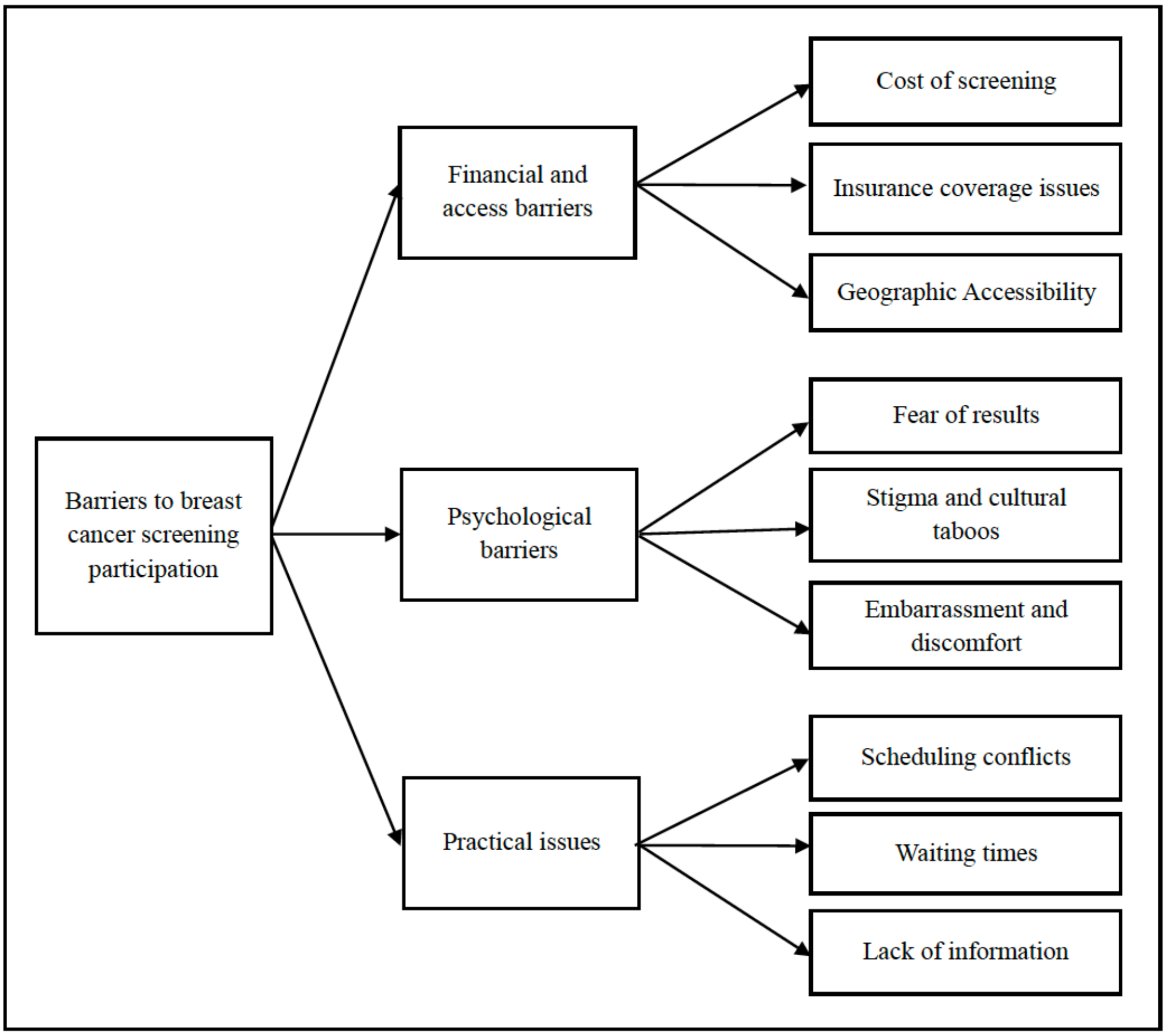

Supplementary File S3. Sociodemographic Characteristics of Cognitive Debriefing Participants (n = 10)

Variable	n (%)
Gender	
Male	2 (20.0)
Female	8 (80.0)
Age (in years)	Median = 33.0 (IQR: 26.8 - 38.5)<br>Min = 24<br>Max = 41
Ethnicity	
Malay	8 (80.0)
Chinese	1 (10.0)
Indian	1 (10.0)
Marital status	
Single / Unmarried	1 (10.0)
Married	8 (80.0)
Divorced	1 (10.0)
Highest education level	
Secondary	5 (50.0)
Tertiary	5 (50.0)
Current employment status	
Unemployed	3 (30.0)
Employed	7 (70.0)
Employment sector	
Government / Semi-Government	3 (30.0)
Private	4 (40.0)
Unemployed	3 (30.0)
Monthly household income (Ringgit Malaysia)	
< 2,500	4 (40.0)
2,500 – 3,169	3 (30.0)
3,970 – 4,849	3 (30.0)
Area of residence	
Urban	10 (100.0)

Supplementary File S4. Sociodemographic Characteristics of Test–Retest Participants (n = 30)

Variable	n (%)
Gender	
Male	10 (33.3)
Female	20 (66.7)
Age (in years)	Median = 23.0 (IQR = 22.0 - 37.3)
	Min = 20
	Max = 56
Ethnicity	
Malay	14 (46.7)
Chinese	8 (26.7)
Indian	6 (20.0)
Others	2 (6.7)
Marital status	
Single / Unmarried	26 (86.7)
Married	4 (13.3)
Highest education level	
Secondary	1 (3.3)
Tertiary	29 (96.7)
Current employment status	
Employed	10 (33.3)
Student	20 (66.7)
Employment sector	
Government / Semi-Government	9 (30.0)
Private	1 (3.3)
Unemployed	20 (66.7)
Monthly household income (Ringgit Malaysia)	
< 2,500	6 (20.0)
2,500 – 3,169	4 (13.3)
3,170 – 3,969	2 (6.7)
3,970 – 4,849	2 (6.7)
4,850 – 5,879	2 (6.7)
5,880 – 7,099	1 (3.3)
7,100 – 8,699	1 (3.3)
8,700 – 10,959	1 (3.3)
10,960 – 15,039	1 (3.3)
> 15,039	10 (33.3)
Area of residence	
Rural	5 (16.7)
Urban	25 (83.3)
